# Natural Antioxidants in Salmon Aquaculture: Processing Fate, Tissue Deposition, and Oxidative Protection

**DOI:** 10.1155/anu/2393517

**Published:** 2025-12-01

**Authors:** Pedro Araujo, Viviana Sarmiento, Odd Elvebø, Rebecca Heavyside, Elisabeth Ødegård, Sandeep Sharma, Kristin Hamre

**Affiliations:** ^1^Feed and Nutrition Research Group, Institute of Marine Research (IMR), Bergen, Norway; ^2^Arctic Feed Ingredients AS, AFI, Steinkjer, Norway; ^3^Nutrition and Formulation R&D, BioMar AS, Trondheim, Norway; ^4^Hamre Aquatic Nutrition, Bergen, Norway

**Keywords:** antioxidants, fish feed, fish tissue, oxidative stress, polyphenols

## Abstract

The use of natural antioxidants (NAOXs) to prevent feed oxidation and reduce oxidative stress in fish is gaining momentum in the aquafeed industry. As sustainable alternatives to synthetic antioxidants like ethoxyquin (banned) and butylated hydroxytoluene (BHT)/butylated hydroxyanisole (BHA) (under scrutiny), NAOX (particularly polyphenols) require assessment for their stability during feed production and their biological effects on fish. This study followed four natural polyphenols (NPs) in a plant-based ingredient, which was then incorporated into a standard feed. The polyphenols were tracked through various processing stages (ingredients, diet mix, extrusion, drying, and storage) and in fish tissues (liver and muscle) using liquid chromatography mass spectrometry. Post-smolt Atlantic salmon were fed diets containing 0.00%, 0.01%, or 0.05% of the polyphenol-rich ingredient for 100 days. NAOX supplementation led to increased levels of astaxanthin, *α*-tocopherol, and *γ*-tocopherol, and reduced thiobarituric acid reactive substance (TBARS) in the feed, indicating improved oxidative stability. In the fish, NAOX had no effect on growth, organ indices, cataracts, or fillet color. However, it significantly reduced malondialdehyde (MDA) levels in liver and muscle and lowered hepatic glutathione (GSH), suggesting improved antioxidant protection. Among the four NPs, only three were consistently detected in fish tissues, especially in the liver. Although extrusion caused substantial losses, the polyphenol profiles in feed and tissue reflected those of the original ingredient. This is the first study to trace NAOXs throughout the entire production chain in salmon aquaculture. The findings support the use of NAOX to enhance feed stability and fish oxidative resilience in a sustainable and effective manner.

## 1. Introduction

Industrial farming may cause high oxidative stress in fish as a result of stressful husbandry practices on farms and factors such as climate change, pollution, suboptimal diet, handling, crowding, hypoxia, and transportation [1, 2]. When it comes to the fish feed, the levels of marine proteins have decreased in favor of plant proteins, accompanied by the reduction/replacement of the fish oil with vegetable oils in the fish diet, both changes for reducing the environmental impact of aquaculture on wild fish stocks [2]. All these conditions may cause stress and oxidation in the fish, affecting their welfare and hence the productivity of the farms [1, 3–11]. For the continued successful contribution of aquaculture to global food security, its development must be based on environmental and economic sustainability. Thus, efforts must be made to increase production whilst protecting fish from the detrimental effects of intensive production practices and challenging conditions.

Synthetic antioxidants, such as ethoxyquin, butylated hydroxytoluene (BHT), and butylated hydroxyanisole (BHA), have been used as feed additives since 2003 (EC No1831/2003) to limit oxidation, thus stabilizing the feed, preventing losses of vitamins and pigments, increasing shelf life, and maintaining a nutritious diet for the animals [12–16]. In 2017, the European Commission (EC No 962/2017) banned ethoxyquin due to its adverse health effects. In addition, it is expected that other synthetic antioxidants will be phased out. Therefore, there is a vital need for suitable, natural alternatives.

Plant-based bioactive compounds are an important source of natural antioxidants (NAOXs) and have been regarded as greener and more environmentally friendly alternatives to synthetic antioxidants [13, 15, 16]. NAOX compounds, such as polyphenols and phenolic diterpenes, are present in plants and algae and can act in defense against stressors such as UV light and reactive species containing nitrogen and oxygen, and also against microorganisms causing diseases [17, 18]. Their antioxidant and organoleptic properties have been acknowledged since ancient times and nowadays studies on these kinds of phytochemical compounds are continuously increasing, especially for their role in preventing feed oxidation and fish oxidative stress and their subsequent impact on fish welfare and farm productivity [18].

There is a myriad of dietary studies on the supplementation of NAOX and their effect on development and welfare of mammals [13, 19, 20]. In contrast, equivalent research on fish is in an early development; however, some increase in publications has been observed in the last years [21–30]. The types of fish studies vary widely. For instance, NAOX had been tested for counteracting negative oxidative stress effects artificially induced by compounds present in fish environment [21, 22], by inadequate conditions of the fish feed [23], as well as by environmental conditions such as water temperature [24], presence of heavy metal contaminants and by bacteria, and other pathogenic microorganisms [25]. Other studies have evaluated their effects on fish development (e.g., growth and weight) and welfare [26–30]. In general, the various studies highlight the importance of including NAOX in fish feed and their positive impact on fish health. Nevertheless, negative effects have been reported when high levels of NAOX were administered to rainbow trout, carp, and seabass [31–34], indicating that the potential benefits of NAOX compounds could be dose dependent.

In addition, for the successful evaluation and control of the inclusion of NAOX into the fish feed, the monitoring of the compounds before/during/after the manufacturing process is needed for determining the real levels that are administered through the diet. Unfortunately, studies covering the concerted analysis of samples during these production stages are rarely considered. The few reported studies have been focused on the protective effect of NAOX against lipid peroxidation in the final extruded fish feed stored at different temperatures by measuring thiobarituric acid reactive substances (TBARSs) [16] and the stability of phenolic compounds during the extrusion process and in the final product using liquid chromatography [35].

Overall, there is a lack of comprehensive studies for the evaluation of NAOX from the biological, chemical, and industrial perspectives, even though it has been suggested that NAOX could outperform the traditional synthetic antioxidants [36]. As legislation and market trends are going toward the use of natural and greener ingredients, detailed and specific studies should be conducted to evaluate the feasibility of the industrial use of NAOX as antioxidant ingredients in fish feed from the economic and functional points of view.

The present study evaluates comprehensively the application of NAOX in Atlantic salmon farming by monitoring its traceability from feed ingredients to fish tissue and evaluating their impact on key chemical/biological factors. Considering that oxidative stress in salmon is particularly pronounced during the first spring in the sea [37, 38], a 3-month tank trial with post-smolts of Atlantic salmon was conducted under simulated natural temperature and photoperiod conditions at Gildeskål (67°N) using three diets supplemented with different levels of antioxidant ingredient (0.00, 0.01, 0.05% w/w). Oxidation and oxidative stress biomarkers were monitored both during the feed manufacturing process and throughout the tank trial. To our knowledge, this is the first study to consider the feed production process as a critical component in the application of NAOX to Atlantic salmon farming. The results are promising, indicating potential benefits for both feed and fish, while underscoring the importance of controlling the production process. This integrated, complex approach is essential for the successful application of NAOX in aquaculture.

## 2. Materials and Methods

### 2.1. Diet

The experimental diets were formulated to meet the nutritional requirements of Atlantic salmon and were produced at BioMar AS pilot plant (Tech Center, Brande, Denmark) using a twin-screw extruder (Clextral BC 45, Firminy, France). The dry feed meal mix was homogenized, preconditioned in an atmospheric conditioner (Clextral, Firminy, France), and subsequently processed in a twin-screw extruder. Postextrusion, the extrudates were dried in a six-layer column dryer (Geelen type). After achieving the desired moisture content, the dried pellets were coated with oil mix in a BES vacuum coater (Brande Entreprenør Service, Brande, Denmark). The NAOX ingredient was provided by Artic Feed Ingredients AS (Steinkjer, Norway) and added to the feed mixture before extrusion, at different inclusion levels (0.0%, 0.01%, and 0.05%). The general composition of the experimental diets is reported in Table 1.

### 2.2. Fish Trials

**Figure 1:**
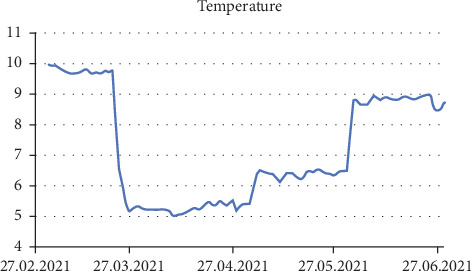
Simulated natural temperature throughout the trial period adapted from photoperiod at GIFAS (67°N).

The fish trials were conducted at Matre Aquaculture Station, Institute of Marine Research, Norway, between March 22nd and June 29th 2021 in nine tanks with photoperiod and temperature at the latitude of Gildeskål Forskningsstasjon AS (GIFAS) commercial sites (67ᵒN) to be able to compare with a previous trial [37]. Each 400 L tank (1 × 1 × 0.4 m^3^) was stocked with 35 post-smolts (initial weight 473 ± 11 g), giving a total of 315 fish across the nine tanks. The temperature was increased from 5 to 9°C (Figure 1) and the photoperiod from 10:14 to 24:0 (L:D) with daily increments. The salinity was 26–29 g L^−1^, and oxygen was kept above 80% saturation. Nine tanks were randomly allocated to the three dietary treatments (Control, NAOX 0.01%, NAOX 0.05%), with three replicate tanks per diet. All fish within a tank received the same diet via automatic feeders.

### 2.3. Sampling Procedure

Different samples were collected before mixing the ingredients described in Table 1 and during the manufacturing process. More specifically, fish meals and fish oil samples, samples before and after the extrusion process, and final diet after the drying process.

The three experimental diets were kept at −20°C in the dark throughout the 3-month trial to preserve their quality and prevent degradation. Daily rations were taken out each day from frozen storage and transferred to the automatic feeders. Samples of the diets were also taken at the end of the experiment.

Fish were sampled at two time points, namely: at the start of the trial (day 1) and after 3 months of feeding. At each time point, 27 fish per dietary treatment (9 fish from each of the three replicate tanks) were euthanized by an overdose of anesthetic Finquel vet. 0.5 g L^−1^. Cataract assessment was performed when fish were under anesthesia and before dissection, using the method of Wall and Bjerkås[39]. Length and weight of the whole body, and weights of viscera and liver were recorded. Liver, muscle, and blood were sampled. Blood was drawn from the caudal vein using a disposable syringe, collected in vacutainers with lithium heparin, and then centrifuged at 3000× *g* for 10 min at 4°C (Jouan MR23i, Saint-Herblain, France). The supernatant was collected and immediately frozen on dry ice. Pooled organ samples were collected from three fish per tank (livers and muscles, respectively) for vitamin C, vitamin E, and astaxanthin analyses and frozen on dry ice. Individual organ samples from an additional three fish were flash frozen in liquid nitrogen for glutathione (GSH)/GSSG, malondialdehyde (MDA), and NAOX analyses. Plasma for analyses of vitamin C and vitamin E was taken from all 9 fish and pooled before analyses. An overview of the process is shown in Figure S1.

### 2.4. Chemical Analysis

Dry matter in the diets was measured after drying at 103°C for 24 h, ash was weighed after burning at 550°C, and lipids after acid extraction by using a method published elsewhere for fish feed [40]. Nitrogen was measured with a nitrogen analyzer (Vario Macro Cube, CN, Elementar Analysensysteme GmbH) according to AOAC official methods of analysis [41], and protein was calculated as N × 6.25. Vitamin C and vitamin E were measured by HPLC according to Mæland and Waagbø [42] and Hamre et al. [43], respectively. The method for astaxanthin analyses is given by Ørnsrud et al. [44]. For the analysis of total and oxidized (GSSG) GSH, supernatants were prepared from tissue sample homogenates using a commercial kit (Prod. No. GT40, Oxford Biomedical Research, Oxford, UK) and then analyzed by a spectrophotometric method at 405 nm in a microplate reader (iEMS Reader Ms., Labsystems, Finland) as previously described [45]. The MDA and TBARS analyses have been described by Hamre et al. [37, 46], respectively. Peroxide value (PV) and anisidine value (AV) analyses were purchased from Nofima AS, Kjerreidviken, Bergen.

### 2.5. Oxidative Stability Measurements

Oxidative stability was measured in hours using an Oxypress apparatus procured by Mikrolab, Denmark. Feed pellets were exposed to 5 bar pure oxygen in an airtight pressure chamber. Heating the chamber to 80°C in an oxygen atmosphere accelerates the lipid peroxidation. The pressure inside the chamber was constantly measured throughout the process. A drop in pressure is an indication of oxygen consumption; thus, oxidation of the sample and antioxidant protection are measured as the time before the sample starts to oxidize. Oxypress feed results are interpreted as the shelf life and values above 30 h are regarded as optimal to fulfill the internal quality criteria of feed production facility (Biomar, Norway).

### 2.6. Analysis of NAOX

Extraction of the fish feed samples containing different levels of antioxidant ingredient (0.00%, 0.01%, and 0.05%) and at different production stages (before extrusion, before drying, and final feed) was carried out by adding 10 mL of methanol to 3 g of sample and stirring magnetically for 25 min. The fish liver and muscle samples (from 537 to 2580 mg) were extracted with 2.5 mL methanol in 5 mL Eppendorf tubes containing glass beads. The tubes were vortex-mixed for 70 min in a Multi-TX5 apparatus (VELP Scientifica, Darmstadt, Germany), centrifuged at 1620 × *g* for 5 min, filtered using 0.45 *µ*m filters, and transferred into amber vials for liquid chromatography tandem mass spectrometry analysis as described by Sarmiento et al. [47, 48]. It is important to note that previous experiments confirmed the presence of gallic acid in the control diet, but not in the antioxidant ingredient. Therefore, gallic acid and four natural polyphenols (NPs) were quantified to evaluate both individual and total contributions of these compounds across the experimental diets. The identities of the four specific polyphenols in the antioxidant ingredient cannot be disclosed due to the proprietary nature of the formulation. These polyphenols are part of a commercially confidential feed mix, and as such, their detailed composition is protected under intellectual property agreements. To maintain the confidentiality of this proprietary formulation while ensuring transparency in the scientific process, these NPs are designated as NP*α*, NP*β*, NP*γ*, and NP*δ* throughout the study. The evaluated polyphenols fall into three chemical classes: phenolic acids (gallic acid), phenylethanoid-type polyphenols (NP*α*), and phenolic diterpenes (NP*β*, NP*γ*, NP*δ*).

### 2.7. Calculations and Statistics

The two-electron half-cell reduction potential of the 2GSH/GSSG redox couple was calculated according to the Nernst equation: Eh = E0´ − RT/nF ln ([GSH]^2^/[GSSG]), where the GSH and GSSG are the concentrations of reduced and oxidized GSH in molar units (M), and Eh and E0´ are the reduction potential of the sample and the standard reduction potential E0´ in volts (V), respectively. The E0´ at pH 7 and 25°C was assumed to be −0.240 V. Statistica data analysis software system (TIBCO Software Inc. 2018, version 13) was used to perform one-way ANOVA, Tukey's post–hoc test, and Kruskal–Wallis multiple comparison test to assess significant differences between pairs of group means and the Levene's test for variance homogeneity.

## 3. Results and Discussion

### 3.1. NAOX in Ingredients and Diets at Different Stages of Production

The results for the different samples are summarized in Table 2. The NAOX ingredient was characterized by the absence of gallic acid and the presence of four NPs, with concentrations ranked as NP*α* > NP*β* > NP*γ* > NP*δ*. In contrast, gallic acid was consistently the major polyphenol in all the control samples at the three steps of the production process (before/after extrusion and final feed) and in all the 0.01% and 0.05% NAOX samples collected after extrusion and in the final feed.

The levels of NP*α* were substantially higher in the NAOX-supplemented feeds before extrusion (4.08 and 8.01 mg/kg for 0.01 and 0.05 % NAOX, respectively) compared to the control (0.65 mg/kg). This indicates that NAOX effectively enriches the feed mix with this specific antioxidant. However, there was a significant reduction in the levels of NP*α* after extrusion (before drying). The 0.05% feed retained higher levels of NP*α* (1.7 mg/g before drying and 1.61 mg/g in the final feed) than the 0.01% feed (0.57 mg/g before drying and 0.44 mg/g in the final feed).

With the exception of gallic acid, important losses in the content of the various polyphenols were observed after the extrusion (before drying) process. Extrusion involves extreme conditions of high temperature and pressure that caused the consumption of an important amount of NAOX compounds, over 80% for NP*α*, NP*β*, NP*γ*, and NP*δ*. However, an interesting fact across the different stages is the preservation of the NAOX ratio 0.05% to 0.01%, which represents the proportional increase in each compound when the concentration of the ingredient is increased. Preserving this ratio suggests that increasing the amount of NAOX consistently enhances the concentration of the measured compounds without unexpected degradation or loss, and that the antioxidant system works effectively across all stages. In addition, the consistency of this ratio allows researchers or feed formulators to predict the levels of different antioxidants in the formulation, making it easier to control the desired levels of bioactive compounds.

In contrast, the increased levels of gallic acid after the extrusion process can be attributed to the presence of propyl gallate in the basal diet (not in the NAOX ingredient). Propyl gallate, an ester of gallic acid and propanol, is commonly added to animal feed to prevent fat and oil oxidation [49]. During the initial mixing phase, prior to extrusion, some free gallic acid may have already been present in the basal diet due to partial hydrolysis of propyl gallate, which was subsequently detected in the NAOX samples. Following the extrusion process, the concentrations of gallic acid in the samples labeled as control, NAOX 0.01%, and NAOX 0.05% increased by 3.3, 3.6, and 3.9 times, respectively. This significant change arose due to the high pressure and temperature experienced during this cooking stage. Statistical comparisons between concentrations in the final feed and post-extrusion samples revealed significant differences (*p*  < 0.05) for gallic acid in the NAOX 0.01% sample, NP*α* in the control sample, and both NP*γ* and NP*δ* across all three samples (control, NAOX 0.01%, and NAOX). These changes in concentration are likely due to a combination of factors, including nutrient degradation, moisture loss, physical alterations, and the well-documented complex equilibrium of compounds in natural ingredients [47, 48, 50]. The higher NP*α* measured in the final control feed versus the post-extrusion sample likely reflects moisture-driven concentration, matrix-dependent extraction/recovery after drying/oil-coating, and potential release from bound precursors during finishing/early storage, rather than deliberate addition.

### 3.2. NAOX in Fish Tissue

The results in Table 3 show the polyphenol content in liver and muscle tissues from the different groups in the tank trial at the start and after 3 months of feeding with the three experimental diets. Among the polyphenols investigated, only NP*β*, NP*γ*, and NP*δ* were detected in the selected tissues. Although gallic acid and NP*α* were the most abundant polyphenols in the final feed, they were not detected in either liver or muscle samples. Notably, in the final feed, NP*β* exhibited a single broad chromatographic peak (~7.00–7.48 min) with a retention time (RT) of 7.24 min. However, in liver and muscle tissue samples, two distinct peaks with identical molecular mass and fragmentation patterns to NP*β* were observed at RTs of 7.02 and 7.17 min. This suggests that the feed contained stereoisomeric forms of NP*β* that co-eluted as one unresolved peak. In the liver and muscle samples, these isomeric forms likely interacted differently with endogenous compounds (e.g., residual lipids or metabolites), resulting in their chromatographic separation into two distinct, sharper peaks. These peaks were combined to estimate the total NP*β* concentration reported in Table 3.

The most salient feature of Table 3 is that NAOX supplementation significantly enhanced the levels of NP*β*, NP*γ*, and NP*δ* in liver. The progressive increase in concentration from Control → NAOX 0.01% → NAOX 0.05% indicates a dose-dependent accumulation of these antioxidants in liver, a key metabolic organ. In muscle tissue, the increase in polyphenol content was more modest but still notable. Overall, NAOX supplementation effectively elevated tissue concentrations of targeted polyphenols, which may improve the oxidative stability of fish tissues, thereby enhancing shelf life and nutritional quality of the final product. Importantly, the liver-to-muscle ratio of total antioxidant compounds remained relatively consistent across treatments, namely: 1.9 (Start), 1.7 (Control), 6.5 (NAOX 0.01%), and 6.4 (NAOX 0.05%). This stability in distribution suggests that despite increased antioxidant intake, the fish maintain a homeostatic regulation of polyphenol allocation between liver and muscle tissues, likely to preserve essential organ function.

Although gallic acid and NP*α* (the latter from the phenylethanoid family) were major polyphenols in the NAOX-enriched feed, their absence in liver and muscle samples is biologically plausible. These compounds are known to be highly metabolically active and are rapidly transformed in the liver into conjugated forms such as glucuronides and sulfates [50, 51]. Additionally, due to their hydrophilic nature, gallic acid and phenylethanoid NP*α* are more likely to be present in kidneys, plasma, or excreted via urine rather than stored in lipid-rich tissues like liver or muscle. For example, a study by DiAngelo and collaborators [52] found that the renal uptake of a phenylethanoid compound was 10 times higher than that of the liver.

### 3.3. Oxidation in Feed Ingredients, Diet Mixes, and Final Feeds

Several parameters were compared to measure oxidation in diets, since it is uncertain which are good indicators. Results are summarized in Table 4. AV and PV were measured in fish oil, fish meal, feed mixes, and final feeds; fresh and after storage for 3 months. The AV value was similar to or lower than the GOED [53] limit of 20 (Global Organization for EPA & DHA Omega-3 s), except in the LT fish meal and in control feed before extrusion and before drying. PV was higher than the GOED's limit of 5 in all cases. It was higher in the fish meals than in the fish oil and the fish meal results were mirrored in the diet mix. The final feed had lower levels of PV than the diet mixes, but the PV increased with storage of the feed. Adding NAOX did not seem to have a positive effect on the AV and PV in the feed.

According to the GOED [53] criteria, fish oil, meals, and the diets at all stages of production were oxidized in the present study. It is difficult to find systematic variation in this data and the NAOX did not seem to affect the results. Furthermore, the methods are commonly used only in oils, and compounds that follow lipid from diets and meals during extraction will probably interfere with the analyses. Therefore, these methods do not seem to give reliable results for these feed matrices.

The concentrations of astaxanthin decreased sharply during extrusion and feed storage, 50% and 65%–75%, respectively, but were relatively stable during feed drying. Astaxanthin levels were numerically higher in the NAOX-supplemented diets than in the control both in the finished feed and after 3 months of storage (e.g., 30 mg/kg DM in NAOX 0.05% vs. 20 mg/kg DM in Control after storage; Table 4), suggesting that NAOX may help limit astaxanthin loss during processing and storage. Nevertheless, NAOX was not sufficient to prevent substantial overall degradation of astaxanthin. The astaxanthin concentration appears to be a good indicator of oxidation in the feed.

TBARS measures mainly MDA, but other compounds also respond to this method, especially in feed. Therefore, HPLC was used for the analysis of fish tissues to separate pure MDA from the other compounds. TBARS was used here as an index of non-volatile aldehydes (e.g., MDA) to compare lipid oxidation status between diet groups. However, it is important to acknowledge that volatile aldehydes are not fully captured by this method and would require complementary analytical approaches. TBARS was sensitive to NAOX during the extrusion step in feed production and during storage of the feed. In the drying process, TBARS values decreased for all diets and the effect of NAOX was not clearly observed. This can be explained by the fact that MDA is volatile, which decreases its value as an indicator of feed oxidation when the feed is exposed to air. However, when the feed is in enclosed containment and not exposed to aeration, TBARS can be a suitable indicator of oxidation. In the analysis after 3 months, TBARS values for the NAOX diets were lower than for the control in a dose-dependent manner. TBARS values were numerically lower in the NAOX-supplemented feeds than in the control after extrusion and during storage, indicating less accumulation of non-volatile aldehydes in those diets. However, because feeds were produced as single batches, this trend cannot be interpreted as statistically significant and should be viewed as indicative rather than conclusive.

Oxypress measures the time it takes before the sample starts to absorb oxygen under oxidative conditions. Theoretically, oxygen consumption starts when the antioxidants have been consumed. The Oxypress results were not affected by NAOX.

The composition of the final feed was analyzed at the beginning and end of the tank trial, and after 3 months of storage (Table 5). Vitamin C and E (*α*-tocopherol) are also antioxidants, but the feed additives are protected against oxidation by addition of inert groups to their active sites and they do not function as antioxidants before the inert groups are removed by digestion in the fish intestine. On the other hand, Gamma-tocopherol is a natural form of vitamin E in the feed coming from the raw materials and is also consumed by oxidation. The decrease in the concentration of gamma-tocopherol was much less than for astaxanthin, but gamma-tocopherol also seemed to be protected by NAOX.

### 3.4. Shelf-Life Considerations Under Commercial Conditions

In the present study, feeds were stored at −20 °C to minimize confounding oxidation during the biology trial. Even under this conservative regime, we observed clear time-dependent changes in oxidative markers over 3 months (e.g., astaxanthin decreased by ~ 65%–75%, while TBARS remained lower in NAOX diets than in the control), indicating that pigment stability is particularly vulnerable and that NAOX partially mitigated lipid oxidation (Tables 4 and 5). The Oxypress test, which accelerates peroxidation at 80°C and 5 bar O_2_, yielded values of ~ 278–289 h for the final feeds, well above the >30 h internal quality criterion used by the production facility, signaling robust baseline resistance to oxidation at the time of manufacture (Table 4). Taken together, these results suggest: (i) the frozen storage facility isolated processing/formulation effects during the trial; (ii) under ambient, commercial storage, oxidative pressures would be higher than in the present trial, especially for pigments; and (iii) NAOX can contribute to preserving feed quality, although it did not prevent major astaxanthin losses under the present conditions. Future work should evaluate NAOX under real-world storage scenarios (ambient temperature, darkness control, oxygen exposure, and humidity) to generate direct shelf-life predictions for farm logistics.

In conclusion, TBARS, astaxanthin, and gamma-tocopherol were good indicators of oxidation in the feed. The data show that the feed oxidizes during production, especially by extrusion, and during storage. The results suggested that NAOX partially protected the feed but did not have the reductive power for total protection of astaxanthin or free *γ*-tocopherol from oxidative degradation, or accumulation of TBARS, under the present conditions.

### 3.5. Effects on the Fish—Feeding Trial

In the present experiment, the fish were held in indoor tanks at Matre Aquaculture Station (IMR) and exposed to simulated natural temperature and photoperiod at GIFAS (67°N) to be able to compare results to a previous study [37]. Cataract increased from March until June, in agreement with Hamre et al. [37] and GSH and vitamin C and E concentrations also changed. The main finding of Hamre et al. [37] was that the increase in fish growth rate during spring and early summer led to more oxidized tissues. The present study hypothesized that this oxidation could be prevented by adding polyphenols as NAOX to the diet.

Fish growth, condition factor, organ indices, and color as measured by salmofan were not significantly affected by the diets (Table 6). The salmofan results agreed with astaxanthin concentrations, which were also unaffected by the diets. The lipid oxidation product, MDA, was similar at start and in the final sample of the control group, both in liver and muscle, but was lowered by ~30% in the 0.05% NAOX group compared to the control.

GSH and GSSG in liver and muscle were higher in the control group at the end of the study than at the start (*p*  < 0.05, Table 7). The *E*_h_ values in liver decreased and became more reduced in this period (*p*  < 0.05) while *E*_h_ in muscle was unchanged. This can be interpreted as the fish increased the GSH concentration and established a more reduced *E*_h_ to protect itself against the increased oxidation in spring [37]. GSH appeared to be metabolized differently in the liver and muscle. In the liver, both GSH and GSSG were lowered by ~25% in response to NAOX (*p*  < 0.01), resulting in a constant redox potential of −0.23 V, that is, constant oxidation state. In the muscle, NAOX addition to the diet resulted in a dose-dependent increase in GSH, whereas GSSG remained constant. This resulted in lowered redox potential from −0.18 to −0.19 (*p*=0.01), that is, the fish muscle was less oxidized. Previous trials have indicated that an early reaction to oxidative stress in fish is to increase the concentration of the endogenous antioxidant GSH [37, 38], The organism regulates the redox potential to modulate metabolic reactions [54]. Therefore, the high GSH concentration in liver may indicate that the control fish were more oxidized than the fish fed NAOX, in line with MDA results, while the different redox potentials in the two tissues indicate that muscle needed to shift the metabolism slightly, while liver had no change in metabolism when exposed to NAOX.

Cataract score was not affected by the diets (Table 7) but increased during the experimental period from 0.9 to 1.4 (*p*  < 0.05), in line with previous studies [37, 38]. There were no effects of diet on vitamin C and vitamin E concentrations in the liver and muscle at the end of the study (Table 8). In plasma, the concentration of vitamin C was higher in fish fed the 0.01% NAOX diet than in control fish, with intermediate concentrations on the fish fed 0.05% NAOX. The diets did not affect the plasma concentrations of vitamin E.

No effects of dietary NAOX were observed on growth, condition factor, body indices, or cataract. Neither were there any effects on vitamin C and E concentrations in plasma, liver, or muscle, except for a minor increase in plasma vitamin C concentration in salmon fed 0.01% NAOX. The higher concentration of astaxanthin in the diet due to addition of NAOX was not reflected in muscle astaxanthin concentrations or in color measured by Salmofan.

## 4. Conclusions

This study presents the first comprehensive investigation into the application of NAOX throughout the entire Atlantic salmon aquaculture production chain, from feed ingredient to fish tissue. It offers a novel and integrated approach by monitoring NAOX compounds during feed processing, tracking their transfer to fish tissues, and simultaneously assessing key biological and chemical indicators of oxidative stress in both feed and fish.

Major antioxidant components in a commercial plant-based ingredient were identified, quantified, and monitored at three critical stages of feed production. The results revealed substantial losses of NAOX compounds during processing, particularly during extrusion, with only about 30% or less of the intended NAOX content ultimately delivered to the fish.

For the first time, the presence of NPs is reported in liver and muscle tissues of Atlantic salmon, marking a significant advancement in our understanding of the uptake and distribution of NAOX in fish.

In the finished feeds and after storage, TBARS values were numerically lower in the NAOX-containing diets than in the control (by ~12%–25%), and the NAOX diets showed higher measured levels of astaxanthin and tocopherols (up to ~ 10% retained after 3 months of storage). However, p-AVs were higher in the NAOX diets than in the control, indicating increased levels of other secondary oxidation products. Because each diet was produced as a single batch and no statistical comparisons can be made, these oxidation markers point in different directions and do not allow us to conclude that NAOX had a consistent protective effect on feed rancidity.

In fish, oxidative stress was mitigated, with more than 25% reduction in MDA in both liver and muscle. Furthermore, GSH levels, typically elevated in response to mild oxidative stress, were lower in the liver and muscle of NAOX-fed salmon, supporting the observed decrease in oxidative pressure indicated by reduced MDA levels.

Under the present conditions, NAOX supplementation improved feed oxidative stability (lower TBARS and better retention of astaxanthin and *γ*-tocopherol during processing/storage) and was associated with lower MDA and altered GSH/GSSG patterns in fish tissues. We observed no effects on growth, condition indices, cataracts, or fillet color. These findings provide practical insight into NAOX use in aquafeeds while indicating that any implications for fish health outcomes require targeted study.

## Figures and Tables

**Table 1:** Chemical composition of experimental diets.

Ingredients (%)	Control	NAOX 0.01%	NAOX 0.05%
Fish meal NA LT^a^	7.50	7.50	7.50
Fish meal NA^b^	7.50	7.50	7.50
Soya SPC^c^	20.00	20.00	20.00
Wheat gluten^d^	12.28	12.28	12.28
Guar meal^e^	10.00	10.00	10.00
Wheat^f^	12.66	12.66	12.66
Fish oil^g^	8.10	8.10	8.10
Rapeseed oil^h^	17.08	17.08	17.08
Vitamin C (added)	0.09	0.09	0.09
Vitamin E (added)	0.01	0.01	0.01
Mono-calcium phosphate (MCP)	2.42	2.42	2.42
Amino acid mix	1.21	1.21	1.21
Premix vitamins, minerals, and other	2.01	2.01	2.01
Natural antioxidant (NAOX)	0.00	0.01	0.05

^a^Pelagia, Norway.

^b^999, Denmark.

^c^Via köster, Brazil.

^d^Aminola, Netherland.

^e^Sunita, India.

^f^Hedegaard, Denmark.

^g^Peru, ED&F Man.

^h^Aminola, Denmark.

**Table 2:** Concentrations of analyzed polyphenols in the NAOX ingredient, feed mix (before and after extrusion), and final feed.

Dietary sample	Gallic acid	NP*α*	NP*β*	NP*γ*	NP*δ*
NAOX ingredient	nd	13.4 ± 0.9	5.0 ± 1.4	2.9 ± 0.4	2.5 ± 0.6
Before extrusion					
Control	1.67 ± 0.27	0.65 ± 0.06	0.14 ± 0.03	0.24 ± 0.02	0.01 ± 0.00
NAOX 0.01%	1.73 ± 0.36	4.08 ± 0.20	0.99 ± 0.15	0.80 ± 0.10	0.25 ± 0.01
NAOX 0.05%	1.73 ± 0.16	8.01 ± 0.80	1.97 ± 0.14	1.65 ± 0.15	0.44 ± 0.07
Ratio 0.05/0.01	1.0	2.0	2.0	2.1	1.8
After extrusion					
Control	5.50 ± 1.60	0.002 ± 0.000	0.02 ± 0.00	0.01 ± 0.00	0.01 ± 0.00
NAOX 0.01%	6.17 ±0 .31	0.57 ± 0.09	0.13 ± 0.01	0.04 ± 0.00	0.09 ± 0.01
NAOX 0.05%	6.78 ± 1.29	1.70 ± 0.19	0.34 ± 0.03	0.043 ± 0.008	0.25±0.04
Ratio 0.05/0.01	1.1	3.0	2.6	1.0	2.9
Final feed					
Control	4.36 ± 0.44	0.29 ± 0.056	0.02 ± 0.00		
NAOX 0.01%	3.24 ± 0.94	0.44 ± 0.06	0.17 ± 0.03	0.22 ± 0.04	0.16 ± 0.02
NAOX 0.05%	4.46 ± 1.03	1.61 ± 0.24	0.48 ± 0.08	0.91 ± 0.13	0.59 ± 0.03
Ratio 0.05/0.01	1.4	3.7	2.8	4.1	3.7

*Note:* Values are expressed as g/kg for the NAOX ingredient and as mg/kg for all other samples.

Abbreviation: nd, not detected.

**Table 3:** Concentrations of polyphenols in fish liver and muscle tissues at the start of the trial and after 3 months of feeding with the three experimental diets.

Tissue type	Diet	Gallic acid	NP*α*	NP*β*	NP*γ*	NP*δ*
Liver	Start	nd	nd	0.051 ± 0.021	0.015 ± 0.009	0.002 ± 0.001
	Control	nd	nd	0.023 ± 0.004	0.004 ± 0.002	0.006 ± 0.003
	NAOX 0.01%	nd	nd	0.077 ± 0.021	0.384 ± 0.142	0.202 ± 0.069
	NAOX 0.05%	nd	nd	0.127 ± 0.022	0.689 ± 0.086	0.329 ± 0.068

Muscle	Start	nd	nd	0.032 ± 0.006	nd	0.003 ± 0.001
	Control	nd	nd	0.019 ± 0.004	nd	0.001 ± 0.001
	NAOX 0.01%	nd	nd	0.046 ± 0.010	0.037 ± 0.012	0.019 ± 0.006
	NAOX 0.05%	nd	nd	0.063 ± 0.013	0.076 ± 0.022	0.040 ± 0.013

*Note:* Values are expressed as mg NAOX/kg of tissue.

Abbreviation: nd, not detected.

**Table 4:** NAOX: oxidative status of raw materials and diets during production and storage.

Sample type	DM (% w/w)	Astaxanthin (mg/kg DM)	TBARS (nmol/g)	Anisidine value	PV (meq/kg lipid)	Lipid (% w/w)	Oxypress (h)
Raw materials							
Fish oil	na	na	na	14	11	na	na
Fish meal NA	na	na	na	19	33	9	na
Fish meal NA LT	na	na	na	88	29	12.4	na

Before extrusion							
Control	93	177	16	47	32	6.2	na
NAOX 0.01%	93	140	16	na	na	na	na
NAOX 0.05%	93	149	16	na	na	na	na

Before drying							
Control	75	65	17	164	27	4.4	na
NAOX 0.01%	75	68	15	na	na	na	na
NAOX 0.05%	75	71	14	na	na	na	na

Final feed							
Control	94	75	8	20	13	31	289
NAOX 0.01%	94	80	6.9	20	11	31	278
NAOX 0.05%	93	87	7.5	21	11	30	289

After 3 months							
Control	94	20	9.4	20	17	31	na
NAOX 0.01%	94	24	8.2	23	13	30	na
NAOX 0.05%	95	30	7.1	26	16	30	na

Abbreviations: DM, dry matter; na, not analyzed; PV, peroxide value; TBARS, thiobarituric acid reactive substance.

**Table 5:** Proximate composition of the final feed formulation analyzed at the start and end of the tank trial, and after 3 months of storage.

Diet	Dry matter (% w/w)	Lipid (% w/w)	Protein (% w/w)	Ash (% w/w)	Vit C (mg/kg)	*α*-Tocopherol (mg/kg)	*γ*-Tocopherol (mg/kg)
Final feed							
Control	94	28	47	6.2	400	310	98
NAOX 0.01%	94	29	44	6.3	440	330	103
NAOX 0.05%	93	29	43	6.2	410	350	104

After 3 months							
Control	94	28	41	6.1	420	290	92
NAOX 0.01%	94	28	42	6.2	450	300	97
NAOX 0.05%	95	28	42	6.3	430	320	99

**Table 6:** Effects of dietary NAOX on growth performance, organ indices, and flesh color in salmon at start and at the end of the experiment.

Group	Weight (g)	Length (cm)	CF	SGR	HSI (%)	Heart (%)	Carcass (%)	Salmofan
Start	444 ± 18	34.7 ± 0.6	1.06 ± 0.02		1.4 ± 0.1	0.13 ± 0.00	91 ± 0.2	20.6 ± 0.2
After 3 months								
Control	931 ± 28	42.0 ± 0.3	1.25 ± 0.02	0.67 ± 0.01	1.1 ± 0.2	0.13 ± 0.02	91 ± 1	22.1 ± 0.2
NAOX 0.01%	925 ± 27	41.9 ± 0.3	1.25 ± 0.02	0.66 ± 0.03	1.2 ± 0.2	0.13 ± 0.01	90 ± 1	22.0 ± 0.2
NAOX 0.05%	894 ± 27	41.4 ± 0.3	1.25 ± 0.02	0.63 ± 0.04	1.1 ± 0.2	0.13 ± 0.02	90 ± 1	21.9 ± 0.2

Abbreviations: CF, condition factor; HIS, hepatosomatic index; SGR, specific growth rate.

**Table 7:** Levels of malondialdehyde (MDA, nmol/g WW), reduced and oxidized glutathione (GSH/GSSG, *μ*M), redox potential (Eh, V), and astaxanthin (mg/kg) in liver and muscle tissues, along with cataract scores in fish at day 1 (start) and after 3 months of feeding (control, NAOX 0.01%, NAOX 0.05%).

Tissue type	Biomarker	Start	Control	NAOX 0.01%	NAOX 0.05%	ANOVA
Liver	MDA	1.27 ± 0.34	1.46 ± 0.23^a^	1.21 ± 0.17^ab^	1.07 ± 0.10^b^	0.002
	GSH	1287 ± 266*⁣*^*∗*^	1924 ± 197^a^	1450 ± 214^b^	1400 ± 132^b^	<10^−4^
	GSSH	5.67 ± 0.90*⁣*^*∗*^	6.9 ± 1.0^a^	4.2 ± 0.4^b^	4.6 ± 0.6^b^	0.0002
	Eh	−0.225 ± 0.004*⁣*^*∗*^	−0.232 ± 0.001	−0.231 ± 0.004	−0.229 ± 0.002	ns
	GSH/GSSH	227.0	278.8	345.2	304.3	

Muscle	MDA	0.37 ± 0.07	0.36 ± 0.07^a^	0.27 ± 0.08^b^	0.25 ± 0.04^b^	0.006
	GSH	55 ± 19*⁣*^*∗*^	79 ± 22^a^	93 ± 18^ab^	120 ± 44^b^	0.02
	GSSH	0.49 ± 0.08*⁣*^*∗*^	0.69 ± 0.08	0.62 ± 0.16	0.74 ± 0.13	ns
	Eh	−0.177 ± 0.010	−0.182 ± 0.007^a^	−0.188 ± 0.002^ab^	−0.191 ± 0.007^b^	0.01
	GSH/GSSH	112.2	114.5	150.0	162.2	

	Astaxanthin	na	2.56 ± 0.75	2.82 ± 0.34	2.87 ± 0.92	ns
	Cataracts	0.89 ± 0.93*⁣*^*∗*^	1.4 ± 1.1	1.7 ± 1.2	1.8 ± 1.4	ns

*Note:* Values with different superscript lowercase letters (a, b) within the same row differ significantly (ANOVA followed by post-hoc multiple comparison test; *p* < 0.05). Values marked with “*⁣*^*∗*^” differ significantly from the Control group (*p* < 0.05).

Abbreviations: na, not analyzed; ns, not significant.

**Table 8:** Concentrations of vitamin C and vitamin E (TOH, mg/kg tissue or mg/L plasma, WW) in liver, muscle, and plasma at the end of the experiment.

Vitamin	Biological sample	Control	NAOX		*p*-Value
			0.01%	0.05%	
C	Liver	247 ± 55	220 ± 10	237 ± 12	ns
	Muscle	39 ± 10	36 ± 7	36 ± 1	ns
	Plasma	40 ± 3^a^	48 ± 3^b^	42 ± 1^ab^	0.02

E (α-tocopherol)	Liver	2013 ± 175	2190 ± 312	2117 ± 423	ns
	Muscle	11 ± 1	12 ± 1	10 ± 2	ns
	Plasma	70 ± 9	61 ± 6	60 ± 4	ns

E (γ-tocopherol)	Liver	133 ± 7	157 ± 8	166 ± 37	ns
	Muscle	3.1 ± 0.4	3.6 ± 0.3	3.2 ± 0.6	ns
	Plasma	11.2 ± 2.4	104 ± 0.4	9.9 ± 0.9	ns

*Note:* Values with different superscript lowercase letters within a row differ significantly (ANOVA followed by post-hoc multiple comparison test, *p* < 0.05).

Abbreviation: ns, not significant.

## Data Availability

All the data are available in the article.

## Supporting Information

Supporting Information Figure S1: Overview of the study design. Adapted from [48]
