# Supplementary material for: Natural Antioxidants in Salmon Aquaculture: Processing Fate, Tissue Deposition, and Oxidative Protection
Source: Aquac Nutr. 2025 Dec 1;2025:2393517. doi: 10.1155/anu/2393517 (PMC12685416; doi:10.1155/anu/2393517)
Supplement: Supporting Information — Figure S1: Overview of the study design. Adapted from [48] [file 2393517.f1.docx]

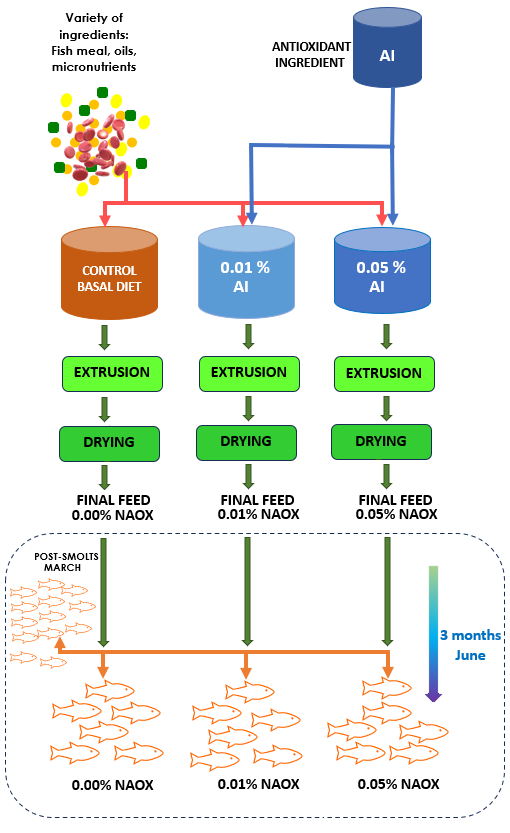


Figure S1: Overview of the study design. A basal diet was supplemented with a NAOX ingredient at three concentrations: 0.00%, 0.01%, and 0.05%. The formulated diets were used in a three-month tank trial with post-smolt Atlantic salmon. Various indicators of oxidation and oxidative stress were measured in both feed and fish before, during, and after the trial. The 0.01% and 0.05% values indicate the intended inclusion levels of the NAOX ingredient at formulation. The actual concentrations of individual polyphenols in the final feeds were quantified by LC–MS/MS and are reported in Table 2 (adapted from Sarmiento’s Master’s thesis [48]).
